# Supplementary material for: Are carbon emissions trading and green financial instruments synergistic? -Comprehensive quantitative research based on content analysis
Source: PLoS One. 2024 Mar 7;19(3):e0298601. doi: 10.1371/journal.pone.0298601 (PMC10919601; doi:10.1371/journal.pone.0298601)
Supplement: S1 Data — (ZIP) [file pone.0298601.s001.zip › Data/地级市Z_F工作报告.zip!/╜¡╦╒/┴1⁄4╘╞╕█╩╨/└┤╫╘╣1⁄2╓┌║┼í╛┬φ┐╦╩2╛▌═°í┐_2010.docx]

各位代表：

现在，我代表市人民政府向大会作工作报告，请予审议，并请各位政协委员和其他列席人员提出意见。

回顾与总结

刚刚过去的2009年，是新世纪以来我市经济社会发展最为困难的一年。受国际金融危机影响，全市保增长保民生保稳定遇到的压力和挑战前所未有。在中共连云港市委的坚强领导下，我们深入学习实践科学发展观，全面把握形势，从容决策应对，团结带领全市人民，抢抓重大历史机遇，统筹推进各项工作，完成了市十二届人大二次会议确定的目标任务。

--经济平稳较快增长。预计实现地区生产总值935亿元，增长13.5%；全社会固定资产投资1000亿元，增长28.6%；社会消费品零售总额369.4亿元，增长19%。三次产业结构调整为16.2:47.3:36.5。

--发展质量逐步提升。完成财政总收入232.3亿元，增长28.6%；其中一般预算收入90.2亿元，增长36.3%。提前实现十一五规划目标。100个工业新增长点项目投产达效，规模以上工业利税、利润分别增长20%、24%。一批重大基础设施开工建设。

--人民生活继续改善。城镇居民人均可支配收入达到16850元，农民人均纯收入6000元，分别增长10.5%、10%。金融机构各项存款余额突破1000亿元，其中城乡居民储蓄存款余额452.9亿元，比年初增加54.5亿元。城镇登记失业率3.1%，居民消费价格总水平下降0.7%。

一年来，我们开展的工作及取得的成效主要有以下几个方面：

一、坚持力保增长促进转型，经济发展实现逆势上扬

积极应对国际金融危机影响，坚定跨越信心，力保强劲增势，千方百计扩大投入、稳定生产、促进消费，推进转变发展方式，提升经济综合实力。

保增长措施有效落实。全面贯彻扩大内需政策，出台稳定工业生产、扶持中小企业、促进外贸稳定系列工作措施。加大项目争取力度，166个项目列入国家扩大内需投资计划，争取中央和省资金10.2亿元。全年争取各类扶持资金42.9亿元。185项亿元以上在建项目加快推进，完成工业投资628亿元，增长25.5%，实现规模以上工业增加值360.2亿元，增长16.5%。建筑业增加值增长20.8%。推动消费扩量升级，形成一批消费亮点。全市新增汽车销售2.8万辆，完成商品房销售514万平方米，分别增长79.1%、130.6%。家电下乡呈现旺势。加大金融支持发展力度，新增各项贷款214亿元，是上年的2.2倍。落实出口退税9.3亿元，增长32.9%。

先进制造业加快成长。坚持调结构与保增长并重，编制实施船舶工业、装备制造等9项产业振兴规划，推动优势产业集聚发展，中材高新硅材料等6个重大产业项目和新能源装备、创新药物等特色产业基地建设进入省双百工程，新医药、新材料、新能源和装备制造业销售收入占规模以上工业比重40%，较上年提高5个百分点。大力发展高新技术产业，恒瑞医药成为国家级创新型试点企业，中复连众3兆瓦海上风电叶片下线，42个产品被认定为省级高新技术产品，新增科技孵化器面积4.5万平方米，实现高新技术产业产值337亿元，增长62.8%，占规模以上工业比重提高4.6个百分点。推进省制造业信息化科技工程示范市建设，制造业信息化应用覆盖率提高10个百分点。汤沟成为中国驰名商标，太阳雨热水器获省出口名牌称号。认真落实节能减排措施，实施热电联产等20项节能降耗重点工程，完成16项污染减排项目，单位地区生产总值能耗下降完成省定目标，化学需氧量削减4580吨，二氧化硫减排完成十一五任务。

高效农业快速发展。加快推进高效农业规模化，新增高效农业面积41.5万亩、高效渔业面积4.2万亩，畜禽规模化养殖比重提高6.5个百分点，成为全国最大的泥鳅养殖出口基地、蝴蝶兰种苗生产基地和省内最大设施渔业基地、鲜切花基地。灌云县、灌南县分别建成全国最大的芦蒿和纯白金针菇生产基地。认真落实强农惠农政策，发放粮食等政策性补助资金4亿元，建成9个国家级高产万亩示范区，粮食总产334.5万吨，连续七年丰产丰收。完成绿化造林24.5万亩，森林覆盖率达19.5%。新增农业利用外资项目20个，农产品出口连续四年居全省前列。农产品质量品牌建设继续加强，申报无公害品牌50个、绿色食品品牌8个、省级名牌农产品2个。东海老淮猪肉通过国家地理标志保护产品认定。

现代服务业继续提升。港口运输在亿吨大港的新起点上增开航线12条、五定班列4趟，实现吞吐量1.14亿吨、集装箱运量303万标箱。科技创意产业园等28个服务业重点项目加快推进，金海国际商务大厦主体封顶，保税物流中心获批建设，全市新发展物流企业310家。实施民航机场国际候机厅改扩建工程，客运量突破30万人次。旅游业实现新发展，东海国际水晶珠宝城成为国家AAAA级景区，新增全国农业旅游示范点3个，境内外游客接待量、旅游总收入分别增长10%、12%。第三代移动通讯服务全面开通。商贸流通业加快提升，家乐福、沃尔玛等世界知名零售企业进驻港城，中翔钢材等一批区域性专业市场投入运营。服务业增加值增长15.2%。

二、坚持抢抓机遇谋划发展，新一轮沿海开发迅速展开

以连云港为龙头的江苏沿海地区发展规划获得国务院批准，港城发展跃上国家战略平台，我们积极放大政策效应，组织动员全市结合实际，扎实推进规划实施。

规划对接落实工作有效开展。建立沿海开发工作推进机制，制定贯彻规划三年行动方案。加强保税港区等重大政策争取，强化沿海开发新优势宣传推介，狠抓重大产业项目招商，迅速形成推进沿海开发的强劲声势。徐圩港区、赣榆港区总体规划通过部省联合审查，灌河口拦门沙治理乘势启动。石化产业基地、大型商品粮储备基地等一批重大项目成功签约，田湾核电5、6号机组获准开展前期工作，华电灌云风力发电、华东物料城、华磁电器等一批项目加快推进，国电联合动力、万吨碳纤维一期工程等产业项目建成投产。

重大基础设施建设全面提速。港口30万吨级航道获准立项，先导试挖工程顺利启动，30万吨原油码头签订合作协议，30万吨矿石码头具备水水中转条件，庙三突堤集装箱泊位实现临时靠泊。主体港集疏运体系不断完善，南疏港道路二期工程基本建成，东疏港高速公路、内河港建设加快推进，开工建设北疏港通道。徐连铁路电气化改造竣工运营。连临高速公路路面基层基本完成，242省道路基贯通，310国道宋跳至大酒壶段建成通车。盐灌船闸通航，盐河航道整治开工。通榆河北延、新沭河治理、大浦河调尾等重点水利工程有效推进，500千伏伊芦变扩建、220千伏龙河、墅港等一批输变电工程建成使用。

沿海生产力布局优化拓展。强势启动徐圩新区开发，编制完成发展战略规划、总体规划、产业启动区和高新区控制性详细规划。226省道北延段和区内徐新路、港前路、徐圩大道等7条道路快速推进，基础设施建设框架全面拉开。申报国家东中西区域合作示范区取得积极进展。建立投融资平台，与多家金融机构开展战略合作，获得银行授信300亿元。同步开展产业项目招商，签订重大产业项目合作协议7项。连云港开发区五园五中心建设加快推进。灌河船舶工业园获批省级特色产业园，两翼临港产业区承载功能不断增强。

三、坚持扩大开放创业创新，跨越发展动力持续增强

立足增创发展新优势，加快重点领域和关键环节改革，推动全民创业，推进开放型经济转型升级，增强经济社会发展活力。

科学发展体制机制加快构建。以学习实践科学发展观活动为引领，制定实施转变发展方式等9个文件，进一步确立科学发展工作导向和激励机制。启动实施新一轮政府机构改革。整合创新领导体制和工作机制，筹建徐圩新区和科教创业园区管委会，顺利完成板浦镇、宁海乡区划调整。深化事业单位改革，实施义务教育学校教师绩效工资。稳步推进基本药物制度试点、社区卫生机构综合配套改革。推进投融资体制创新，完善投融资平台建设，新海连公司成功发行15亿元企业债券，港口股份获准发债6.5亿元。市财政投入资金2000万元，大力实施创业创新领军人才集聚工程。采取降低出资注册门槛、扶持创业载体、成立县乡创业指导服务中心等多项措施，大力促进全民创业。积极扶持中小企业发展，帮助1300多家中小企业融资30亿元。加大小额担保贷款发放力度，实施春风进万家创业助贷工程。加快壮大民营经济，新增私营企业6000户、个体工商户3万户，民营经济增加值增长15%。

开放型经济发展逆势奋进。强化招商引资工作，积极拓展与海内外大企业合资合作，在境内外密集举办投资说明会，利用台湾江苏周等各类经贸平台，广泛集聚外来资源，全市注册外资实际到账10.3亿美元，内联客方到位资金370亿元。扶持外贸企业发展，调整出口产品和市场结构，完成出口19.5亿美元。创新外派劳务市场管理机制。积极支持企业走出去发展，新增境外注册商标企业6家。实施园区开发提升计划，出口加工区拓展了保税物流功能、二期建设通过海关验收，省级以上开发区固定资产投资增幅高于全社会3.3个百分点，规模以上工业销售收入增长41.3%，占全市比重68.6%。连云港开发区在国家级开发区排名中较上年上升1位。推进与长三角、珠三角、中西部地区互动合作发展，与上海、郑州、西安等重要城市建立战略合作机制，连云港-深圳新型产业园签约。提升南北挂钩协作水平，连云港开发区江宁工业园、灌云开发区被批准为省南北挂钩共建园区。

四、坚持统筹推进加快建设，城乡一体化发展取得新进展

围绕建设国际性海滨城市目标，加速推进城市化，加快建设新农村，着力构建以城带乡、城乡互动发展新格局。

特大城市框架进一步拉开。新一轮城市发展总体规划获批实施，编制完成城乡统筹、城市生态系统等专项规划20项。连云新城抛填形成陆域10平方公里，起步区胜利湖、海州湾商务中心等重点项目主体完工。科教创业园区、孔望山等片区开发快速推进，中心城市建成区面积突破100平方公里，城市化率达43.2%。城镇发展体系不断完善，赣榆城区加快向沿海拓展，东海、灌云、灌南县城规模扩大，新增市级城市化示范镇4个，燕尾港海滨新城建设步伐加快，温泉镇成功创建省级园林小城镇。

中心城市功能不断提升。东部城区中央商务区建设加快推进，海棠路立交、铁路连云港东站等重点工程竣工使用，新东方供水加压站主体工程竣工。新海城区开工建设苏宁广场等重点项目，完成旧城拆迁改造63.5万平方米。新建续建城市道路18条，城市路网加快完善。发行城市公交IC卡，增开和调整优化公交线路8条。整治刷新城市绿化景观，青年公园开园，孔望山生态园、东盐河景观带一期工程主体完工，开工建设北固山森林公园和海滨公园改造工程，城市人均公共绿地达11平方米。新增自来水干线管道30公里、集中供热面积50万平方米、管道燃气用户9000户，完成人防工程13.6万平方米。数字化城管建设取得积极进展。环境质量持续改善，垃圾焚烧热电厂基本建成，刘湾垃圾填埋场建设加快推进，城市污水处理率达79.3%，市区空气质量优良率93%，公众环境满意率85.6%，提高1个百分点，25项指标达到国家环保模范城创建考核标准。

新农村建设扎实开展。完成15个乡镇总体规划、390个新农村建设规划修编，建设康居示范村30个，开展19个省级村庄环境整治试点。完成182条河道疏浚，新改建排灌站174座，改造中低产田15.7万亩，复垦新增耕地近2万亩。建成农村公路520公里、桥梁130座，城乡客运一体化覆盖率达81.3%，行政村班车通达率99.2%。新改建村卫生室350个，增加农村户用沼气1.5万户，完成改厕3万座。新发展农民合作经济组织540家、土地股份合作社31家，新增土地流转面积13.5万亩，增加集体收入过20万元村73个。农村日用消费品、农资乡级农家店覆盖面达100%。

五、坚持经济社会协调发展，社会建设不断加强

在加快经济发展的同时，大力发展社会事业，深化城市文明创建，维护社会稳定，不断强化和谐社会基础。

科技教育加快发展。深入实施创新驱动战略，积极推动高等院校、科研院所来连兴办研究院。与南京大学合作建设国家重点实验室分室和高新技术研究院，引进南京工业大学筹建海洋工程研究院、江苏大学成立东海机械汽配研究院，与河海大学签订科技合作协议。和东南大学合作组建省高新技术研究重点实验室。江苏海洋资源开发研究院建设加快推进。中科院能源动力研发中心一期工程基本建成，标志着我市拥有了国际先进水平的研发平台。新增省级博士后科研工作站9家、企业院士工作站2家。新上省以上科技计划项目126项。省硅产品检测中心、高性能纤维产品检测中心成功落户。全市专利申请量1500件、授权350件。加大义务教育投入，落实义务教育免费政策，资助贫困生8600万元，启动实施中小学校舍安全工程。促进教育均衡协调发展，开工建设东部城区四星级高中、市未成年人素质教育基地，连云港开发区三星级高中建成招生，苍梧小学主体封顶。全市学前教育毛入学率94.4%，小学、初中入学率基本100%，高中段毛入学率95.1%。大力发展职业技术教育，创建省三星级以上中等职业学校8所、省级示范专业9个、国家级实训基地1个。地方高等教育发展加快，连云港师专、工贸高职校迁入新址。

各项社会事业协调推进。医疗卫生服务体系不断完善，市一院综合楼主体封顶，建成新传染病医院。启动县级中医院、乡镇卫生院、城市社区卫生服务中心建设项目17个，农村社区卫生服务站标准化率87%，城市社区卫生服务中心全部纳入医保定点。新型农村合作医疗参合率达99.5%。疾病防控、卫生监督能力不断增强，高度重视甲型流感疫情防控，为重点人群免费接种疫苗12.5万份，传染病发病率低于全省平均水平。人口和计划生育工作继续强化，人口自然增长率6.9‰，计划生育率90%以上。公共文化服务体系不断完善，开工建设市文化创意产业园、广播影视文化城、东部城区文化创业中心，在苏北率先实现村文化室、农家书屋全覆盖。东海县被评为全国文化先进县，灌南县成功举办国际魔术交流活动。完成城区数字电视整体转换，有线电视入户率72%。群众性体育运动广泛开展，灌云县等8家单位被国家体育总局授予群众体育工作先进单位。我市籍运动员获得十一届全运会2金、4银、2铜的好成绩。母婴安康工程、妇女活动中心等实事项目扎实推进，开工革命纪念馆新馆及陇海公寓复建工程，市气象防灾减灾指挥中心、美术馆建成使用，完成海清寺一期工程。全民国防教育、民兵预备役和双拥优抚工作进一步加强。全面完成经济普查。档案、外事、侨务、对台工作、防震减灾、红十字、老区开发、关心下一代和光彩事业、慈善事业取得新成绩。

精神文明建设和社会管理取得新成效。开展庆祝新中国成立60周年系列活动，组织参加奋进的江苏大型成就展，举办放歌港城歌咏比赛、文化博览会等群众性文化活动500余场。深入开展文明城市创建，获全国未成年人思想道德建设工作先进城市称号。对口援助四川什地镇灾后重建完成投资1.8亿元。加强基层民主政治建设，57个社区和街道受到国家表彰。深入推进法治建设、平安创建，灌南县被评为全国平安建设先进县。推进基层信访基础达标工程，深化大调解工作机制，成立市区医患纠纷调解中心。加强社会治安综合治理，开展汽车、摩托车、电动自行车三车被盗断流和酒后驾车专项整治行动，道路交通事故下降5.5%。安全生产形势保持稳定，连续五年获省考核优秀。严格食品药品监管，开展打击违法添加非食用物质和食品添加剂专项整治，药品生产经营环节监督率100%，零售企业远程监控率95%。加强应急管理工作，神盾2009国家核应急演习圆满完成。

六、坚持富民惠民改善民生，群众利益得到保障

注重以人为本，践行发展为民，认真解决群众切身利益问题，努力提高城乡居民生活水平。

城乡就业规模进一步扩大。大力推进创业带动就业，制定稳定就业和促进创业工作实施意见，落实财税缓征等政策措施，积极帮扶困难企业稳定就业。开展充分就业社区创建和系列就业促进活动，投资1.4亿元建设公共实训鉴定基地，建立毕业生见习基地，多渠道扶持大中专毕业生、下岗失业人员就业。完成职业技能培训24.6万人次，新增城镇就业6.7万人、农村劳动力转移8万人，实现下岗失业人员再就业2.4万人，为2.5万名就业困难对象和灵活就业人员办理社会保险补贴。保持城镇零就业家庭动态为零。

社会保障能力进一步提升。扩大社会保障覆盖面，实现社会保险市区统筹，城乡基本社会保险覆盖率达93.7%。提高11万名企业退休人员养老金标准，人均每月增加128元，推行新型农村养老保险制度，新增参保人员11.4万人。开通城镇职工医疗保险省内异地就医结算，组织20万城镇医保参保人员免费健康检查，积极资助城乡困难群众参加医疗保险。加强被征地农民基本生活保障，新增失地农民全部纳入社会保障。增加城乡低保9313人，发放低保金1.5亿元。市社会福利中心建设、老年大学搬迁改造取得积极进展。农村五保对象集中供养率达70%，提高4.3个百分点。

发展惠民力度进一步加大。优化公共财政支出结构，提高公共产品和公共服务供给能力，民生投入占财政支出比重提高4.8个百分点。加快推进蔷薇河沿线排污截流整治，建成水质自动监测站，竣工大圣湖应急供水工程，启动市区自来水水质深度处理项目，大力实施农村饮用水安全工程，完成60万人改水任务，城乡群众饮用水安全保障进一步增强。加强保障性住房建设，竣工经济适用房22万平方米，建设限价房408套、廉租房880套，发放廉租住房租赁补贴1795户，提供人才安置房100套。发放城镇职工住房公积金贷款18亿元。加大便民工程建设力度，修建便民道路30条，整治人行道板5万平方米，新装小街巷路灯1000盏。加强扶弱助残工作，扎实推进脱贫攻坚工程，帮助农村12.5万贫困人口脱贫。

一年来，我们深入学习实践科学发展观，推进转变政府职能，提升行政效能，建立了行政决策专家咨询、重大决策公示等制度，推进行政权力网上公开透明运行、削减行政权力603项，开通12345政府服务热线，建立行政审批网上平台，停止和减免征收行政事业性、服务性收费71项。坚持依法行政，自觉接受人大及其常委会的法律监督、工作监督和人民政协民主监督，认真办理集中式饮用水源地保护议案，办结人大代表建议135件、政协委员提案421件。综合运用信访、调解、行政复议等多种方式，妥善化解社会矛盾。强化审计监督和行政监察，推行行政问责制，加强廉政建设，改进机关作风，优化发展环境，服务科学发展、跨越发展水平有效提高。

回顾过去一年工作，在推进科学发展、跨越发展的征程上，我们迎难而上、砥砺奋进、力克时艰，有效抵御了国际金融危机冲击，完成了保增长促跨越年度任务，巩固发展了加速崛起的良好态势。更加令人欣慰的是，我们不仅在经历金融危机严峻挑战和重大考验中收获了丰硕发展成果，而且提高了在复杂环境中组织开展工作的能力，积累了在创新思路中破解发展难题的经验。成绩来之不易！这是中共连云港市委正确领导、全市人民团结奋斗、社会各界共同努力的结果。在此，我代表市人民政府，向辛勤工作在各条战线的广大干部群众致以崇高的敬意！向大力支持政府工作的人大代表、政协委员、各民主党派、工商联和人民团体，向为连云港改革发展稳定作出积极贡献的在连部省单位、驻军部队、武警官兵和公安干警，向所有关心支持连云港建设的海内外朋友，表示衷心的感谢！

在肯定成绩的同时，我们也清醒地看到，全市经济社会发展还面临不少困难，存在一些问题。一是产业经济实力还不强，资源环境保护、节能减排约束压力加大。二是重大项目建设、高端人才培养引进等发展制约需要进一步创新思路、大力破解。三是中心城市承载和辐射功能还不强，城市化进程需要进一步加快。四是民生事业满足群众需求还有不小差距。同时，政府自身建设还存在一些薄弱环节，公共服务能力需要进一步增强。对这些问题，我们将高度重视，采取有效措施，切实加以解决。

形势与任务

各位代表，连云港发展正站在新的历史起点上，国家对新一轮沿海地区发展作出重大部署，以连云港为龙头的江苏沿海地区成为国家战略规划的重点发展区域，为我们在更高平台上推进科学发展、跨越发展提供了历史性机遇。连云港发展正处于加快跨越的关键阶段，中央从区域协调发展的战略高度，赋予我市重要使命，全市发展已经进入工业化、城市化加速期，多年积累的发展基础更加坚实，长期集聚的发展潜能蓄势迸发，为我们实现进位争先、崛起振兴提供了强大动力。连云港发展正迎来更加有利的政策支持，宏观经济政策保持连续性稳定性，国家部委支持长三角和江苏沿海地区发展的政策措施正在陆续制订，省委、省政府出台促进沿海开发若干政策意见，为我们会战沿海、昂起龙头提供了良好条件。尽管当前还面临世界经济复苏缓慢曲折、国内经济运行新老矛盾交织的复杂环境，面临新一轮区域发展竞争压力日益增大的艰难挑战，只要我们坚定信心、抢抓机遇、乘势而上，充分利用有利条件，调动积极因素，变压力为动力，就一定能够不断夺取经济社会发展新胜利，书写后发先至的时代新篇章！

根据省市实施沿海开发规划总体部署，从现在起到2012年，是我市立足国家战略平台，推进落实沿海开发规划实现重点突破、全面提速的关键三年。我们将坚持以港口为龙头带动、产业为主攻方向、城市为有力依托、基础设施为坚实支撑、城乡统筹为战略取向的基本策略，持续兴起沿海开发热潮，加快建设大港口、培育大产业、服务大腹地、实现大发展。

综合实力实现跨越提升。到2012年，全市地区生产总值达到1600亿元，力争1800亿元；人均地区生产总值达到35000元，力争40000元。财政收入达到400亿元，其中一般预算收入超过150亿元。累计完成全社会固定资产投资3500亿元。连云港开发区进入国家级开发区综合实力20强。四县力争进入全国百强县行列。

港口发展实现跨越提升。加快建设一体两翼组合大港，建成连云港区25万吨级航道、徐圩港区10万吨级航道，建设赣榆港区5万吨级航道，实施灌河口航道口门整治工程；建设30万吨原油码头、大堤作业区集装箱泊位及赣榆、灌河港区一批万吨级泊位。完善集疏运体系，建成东疏港、北疏港高速公路和通榆河疏港航道，建设盐河、灌河等内河航道，建成连盐铁路，力争空港成为一类开放口岸。完善口岸大通关体系，加快腹地无水港建设，建成运营保税物流中心，争取设立保税港区。港口吞吐能力达到1.6亿吨、集装箱600万标箱。基本建成集装箱干线大港和区域性国际物流中心，新亚欧大陆桥东桥头堡功能显著增强。

产业培育实现跨越提升。大力实施现代农业、新型工业、现代服务业振兴工程。加快建设一批现代农业产业园，打造全国沿海现代农业和新农村建设示范区。积极发展战略性新兴产业，改造提升传统产业，加快培育高新技术产业、临港基础工业、现代服务业三大千亿产业板块，争取大型石化、清洁能源、新型钢铁等项目纳入国家十二五规划并开工建设，努力打造全国沿海新型工业基地。全市规模以上工业销售收入达3000亿元，新医药、新材料、新能源和装备制造业比重超过50%。着力建设区域性航运、物流、商务、金融、信息和旅游中心，推进现代服务业集聚发展。初步形成支撑跨越发展的新型产业体系。

城市建设实现跨越提升。按照一心三极规划布局，加快推进东部城区北拓南延、新海城区互连东进、赣榆城区向中心城区汇聚。实施重点片区提升工程，快速推进连云新城开发，起步区初具规模，基本建成科教创业园区和孔望山片区，加快建设徐圩新区、赣榆海滨新区，推进实施东海西双湖、灌云燕尾港、灌南灌河口半岛新区开发建设。中心城市人口达到120万人，城市化率达50%，四县县城人口均达20万人以上。强力推进国家环保模范城、园林城、卫生城和文明城市创建工作，初步构建现代化的特大城市、富有魅力的国际性海滨城市。

转型升级实现跨越提升。加快建设创新型城市，大力发展循环经济、低碳经济，推动经济结构优化升级，高效农业占耕地面积比重提高到35%以上，高新技术产业产值年均增长30%以上，三次产业结构调整为10:54:36。提升科技创新能力，新医药、新材料、新能源产业研发投入占销售收入比重提高3个百分点，全社会研发投入占地区生产总值比重提高到1.5%，科技进步贡献率达45%以上。加强生态建设和环境保护，提高节约集约发展水平，节能减排达到国家要求，环境质量综合指数保持在85以上。

和谐构建实现跨越提升。到2012年，城镇居民人均可支配收入超过22000元，力争25000元，农民人均纯收入超过8000元，力争9000元，全面完成脱贫攻坚目标。社会保障体系进一步完善，城镇基本养老、基本医疗和失业三大保险覆盖面均达到95%以上，新型农村合作医疗应保尽保。基本实现低保家庭住得上廉租房，低收入家庭住得起经济适用房，新就业人员租得起住房。平安建设深入开展，公众安全感进一步增强，社会事业全面进步，城乡文明程度不断提高，和谐稳定的局面更加巩固。

2010年是全面推进落实沿海开发规划的第一年，也是完成十一五规划、编制十二五规划的关键之年。根据中央、省经济工作会议和市委十届九次全会部署，今年政府工作总的要求是：认真贯彻党的十七大、十七届四中全会精神，深入实践科学发展观，围绕高扬创新大旗、推动全面跨越年度工作主题，把大力推进沿海开发作为首要任务，把顺利完成十一五规划作为决胜目标，加快创新发展，深化改革开放，推进结构调整，促进转型升级，坚持城乡统筹，不断改善民生，保障和谐稳定，奋力开创经济社会科学发展、跨越发展新局面。

主要预期目标是：地区生产总值增长14%，财政一般预算收入增长20%，完成全社会固定资产投资1200亿元，实现注册外资实际到帐10亿美元，引进内联客方到位资金400亿元。社会消费品零售总额增长16%，城镇居民人均可支配收入增长11%，农民人均纯收入增长11%，城镇登记失业率控制在3.6%，居民消费价格涨幅不高于省控标准，节能减排完成省下达任务。这些指标是根据上级精神和我市实际情况确定的，统筹考虑了有利条件和不利因素，兼顾了需要和可能。我们将确保实现，力争完成得更好。

工作与措施

新的一年，面对新形势新任务，我们将进一步解放思想、开拓进取，强化措施、扎实工作，奋力在沿海开发上重点突破，在创新发展上大力推进，在转型升级上积极作为，在城市建设上加快提升，在要素集聚上广泛吸纳，在服务型政府塑造上着力强化，为打造江苏沿海开发龙头奠定坚实基础。政府工作重点抓好以下七个方面：

一、搏取良好开局，重抓以项目推进为主线的沿海开发建设

加快重大基础设施建设，打开沿海开发新局面。坚持把基础设施工程作为推进沿海开发的有效抓手，快速推进港口30万吨级航道建设，开展30万吨原油码头前期工作，30万吨矿石码头、庙三突堤5个集装箱泊位投入运营，建设氧化铝、液体散货泊位，加快旗台防波堤、大堤作业区和徐圩港区10万吨级航道建设，推进实施赣榆港区5万吨级航道、灌河口航道口门整治和码头泊位工程。加快完善集疏运体系，建成东疏港道路，竣工疏港航道和内河港一期工程，推进北疏港通道和盐河航道整治工程建设。提升口岸服务功能，建设一站式服务中心，完善电子口岸信息平台，建设煤炭、矿石等中转配送基地，扩大港口与陆桥沿线、内陆腹地的区港联动。扎实推进连盐铁路建设，争取连宁铁路开工，完成机场国际候机厅扩建和区域性大型机场选址，提升交通枢纽地位。加快实施新沭河治理、大浦河调尾、连云新城挡潮闸等水利项目，完成通榆河北延送水工程。推进220千伏临海、灌河等输变电工程。

重抓徐圩新区开发建设，打造沿海开发新平台。坚持港产城一体化开发，按照基础设施适度超前、产业项目加快推进的要求，整合徐圩新区发展资源，加快云湖核心区、产业启动区和产业服务中心等重点工程建设，加快配套交通、电力、给排水、通讯、水利等基础设施，建设与主城区连接的快速通道。建立共建共用机制，制定激励扶持政策，加快人才、技术、资金和项目集聚，积极承接临港重大项目。完成固定资产投入30亿元。

推进重大产业项目建设，展现沿海开发新成效。开工建设新海发电公司超超临界机组，加快推进华电风力发电、田湾核电5、6号机组扩建工程，启动实施连云港碱厂产业结构调整项目，确保新海石化二期、杰孚特重工、离子膜氯碱等50项重点项目竣工投产。跟踪落实6个省级重大产业项目和31个重大签约项目，积极推进日本生态产业园、高新化纤产业园、盐化工等重大项目。加快完善连云港开发区五园五中心、板桥盐化工集中区、连云港化学工业园和柘汪、燕尾等临港产业园区承载功能，优化空间布局，提升发展层次。

强化政策对接事项争取，增强沿海开发新动力。扎实推进　六六六工程，重点突破十大争取推进事项。认真对接省沿海开发实施意见和分类规划，制订实施综合交通、工业布局等13项具体行动方案。确保石油储备、大型石化基地、新型钢铁、徐连客运专线等一批重大项目进入国家和省十二五规划，力争早日开工建设。推进国家东中西区域合作示范区和保税港区争取工作。加大配套政策争取力度，积极推动国家部委和省有关方面，细化完善沿海开发扶持政策，争取国家和省在城市建设、产业发展、投融资、资源环境、开放合作等方面的更大支持。

二、促进转型升级，提升以质量效益为内涵的经济建设

加快工业经济结构调整。突出新型主导产业培育，以临港基础工业和高新技术产业为重点，加快建设一批带动产业结构优化升级、提升综合竞争力的重点项目，坚持高端切入，积极发展清洁能源、生态环保、生物技术等战略性新兴产业，推动总量扩张与转型调整互动并进。完成全社会工业投资750亿元，确保康缘药业中药注射剂、晶海洋半导体等100个新增长点项目投产达效，规模以上工业增加值增长15.5%。突出特色产业板块打造，实施千亿产业培植计划，加快推进恒瑞豪森新医药产业园、正大天晴新区制剂、博宇船用设备、碳纤维二期、多晶硅等一批重大项目，新医药、新材料、新能源产业产值均突破150亿元，完成装备制造业产值300亿元。大力推进工业集聚发展，省级以上开发区工业销售收入占全市比重提高5个百分点。

推进现代服务业集聚发展。围绕提升功能、打造特色，重点抓好现代服务业集聚区和33个重点项目建设。加快发展现代物流业，推进金港湾国际物流园建设，新建一批无水港，增开五定班列，增辟内外贸航线，启动建设航运交易市场，完成港口吞吐量1.2亿吨，集装箱运量360万标箱。空港具备开放条件，开通境外包机航线，民航客运量超过35万人次。加快区域性国际商务中心建设，积极发展服务外包，引办、承接大型商务会展活动，大力发展研发设计、信息服务、市场中介等生产性服务业。着力发展金融产业，吸引股份制商业银行、外资银行来连开办分支机构，积极筹建地方性银行。推进文化产业发展，加快文化创意产业园建设。加大旅游资源整合力度，加强花果山旅游区开发，争取启动大型主题游乐项目，培育一批特色农业旅游项目，实施旅游年票制度，游客接待量、旅游总收入分别增长12%和15%。加快推进华东物料城、义乌小商品城等重点商贸项目，做大区域性专业市场，扩大社区商业、家政服务等生活消费。服务业增加值增长15%。

加强技术创新能力建设。大力发展创新型经济，推进创新型城市建设，进一步激发全社会创新创造的热情和活力。今年公共财政支持创新力度进一步加大，市财政安排科技进步和创新投入突破1亿元，增长74.5%。加快实施主体培育、平台建设、成果转化三项工程，积极推动企业自主创新，培育高新技术企业30家、高新技术产品50个、信息化科技工程示范企业10家，高新技术产业产值达到430亿元。加快打造创新平台，中科院能源动力研发中心形成研发能力，南京大学连云港高新技术研究院、南京工业大学连云港海洋工程研究院等投入运行，扎实推进硅产品、高性能纤维和风力叶片3个检测中心建设，建立大学科技园、重点实验室、工程技术研究中心、企业院士工作站等研发机构30个，培育农业科技园区10个，新增科技孵化器面积3万平方米。促进产学研联合，推进创新成果转化，组织实施重大科技成果转化项目10项。加强知识产权保护，全市专利申请量1600件、授权量400件。实施人才强市工程，培养引进一批领军型人才。加大品牌培育力度，争创省以上名牌10个。促进企业标准化建设，采用国际标准15项。

推动资源环境持续发展。落实最严格的土地管理制度，坚持集约节约利用土地资源，提高土地投入强度和产出效率，保障重点项目、民生工程建设用地。扎实推进节能减排，严格控制高耗能、高污染项目，重点实施20项节能技术改造项目，认真开展建筑节能标准设计试点，建设低能耗和绿色建筑示范工程，新建建筑执行节能标准比例达到90%。积极发展低碳经济，大力推行清洁生产和资源综合利用，开发太阳能、沼气等清洁能源项目，推广秸秆高效利用技术和生态种养殖模式，发展绿色食品生产和加工业。加强生态建设，推进实施临洪河口等湿地保护，加快建设绿色连云港，大力开展绿化造林，森林覆盖率超过20%。突出抓好水源保护，加强海洋开发管理，保护海洋环境。

三、打造宜居品牌，推进以特大城市为目标的城市建设

建设特大城市，构建新型城镇体系。坚持沿海拓展、组团开发、滚动推进，加快构筑特大城市框架。完成城乡建设投入190亿元，城市化率超过45%，中心城市建成区面积超过120平方公里。连云新城加快推进陆域形成工程，起步区基础设施配套完善，东哨西墅片区展现新形象。加快科教创业园区、孔望山片区等重点区域开发，启动猴嘴片区改造。完善城市外部交通，建成连临高速，加快204、226、242等国省道建设，推进实施临海高等级公路。新建改建凌州东路等城市道路46条，建成花果山大道猴嘴立交，完善连接各城区的快速交通网。大力推进新型城镇化，加快人口向城镇集中、居住向社区集中、土地向规模经营集中，促进四县县城向中等城市发展，完成总体规划修编，加强重点中心镇、临海城镇、特色城镇建设，着力构建沿海沿东陇海线城镇发展轴，形成交通便捷、结构合理、功能互补的区域城镇体系。

彰显地方特色，提升城市建设品味。突出山海特色，凝结文化气质，努力打造个性鲜明的城市生态人文景观。大力推进山海景观轴、滨海休闲带建设，建成使用金海国际商务大厦、金港湾商务大厦等一批重点项目。精心打造城市亮点，改造刷新海棠路、大港路、海连路沿线环境，实施新海城区出入口等一批节点区域整治工程，建设猴嘴文化公园，启动玉带河景观带综合整治。积极培育城市核心商圈，推进苏宁广场、家乐福、沃尔玛等现代城市商业综合体建设，提升陇海步行街、万润商业街、盐河南路餐饮街、墟沟海鲜街等特色街区品位。改善城市窗口形象，完成新浦客运总站扩建，改造苏欣、捷安等汽车客运站。大力推进南极北路、锦屏棚户区改造，完成拆迁改造面积60万平方米，进一步改善城市居住条件。

完善配套功能，塑造城市宜居环境。加大新一轮四城同创推进力度，加快提升城市对人口、产业承载和服务能力。开工建设南城、板桥污水处理厂，续建6座污水提升泵站，新增城市污水管网15公里。完成自来水深度处理工程，新改建自来水管道20公里。新增管道天然气用户8000户、集中供热30万平方米。建成刘湾垃圾填埋场一期工程。实施一批城建惠民项目，深化社区街巷改造。推进城市立体绿化，新增绿地220万平方米，城市绿化覆盖率达到42%。开通运行数字化城市管理系统。完善社区服务设施布局，创新物业管理模式，积极打造市民10分钟生活圈，提升城市居住适宜度。

四、推进城乡统筹，加快以富民增收为核心的新农村建设

进一步构建现代农业体系。突出生态、高效、外向发展方向，提升农业设施化水平，建设国家大型商品粮基地、高效特色农业基地和生态观光农业基地。推动结构优化升级，抓好现代农业示范园区、千亩连片设施农业、粮食高产创建等工程，重点培育优质粮油、设施蔬菜、畜禽养殖、食用菌和花卉苗木五大主导产业，新增高效农业30万亩，其中设施农业15万亩，发展高效渔业10万亩、工厂化养殖10万平方米，畜禽规模养殖比重提高5个百分点。增强龙头带动能力，鼓励农产品精深加工，发展壮大20家龙头企业，新办农产品加工企业100家。加快发展外向农业，实施出口振兴计划，农产品出口保持全省领先水平。编制城郊农业发展规划，打造生态、观光、休闲农业品牌。培育创新支撑体系，加强农业科技攻关，加快引进推广农业新品种、新技术、新装备，高度重视农产品质量安全，提高无公害、绿色和有机农产品比重。

进一步改善农村生产生活条件。落实强农惠农政策，加大三农投入。扎实推进新一轮农村实事，加强基础设施建设，建成农村公路200公里，完成185条河道疏浚和1700个河塘整治，继续实施农村饮用水安全工程，完成42万人改水任务。加快农业资源开发，改造中低产田15万亩，提高农业机械化水平，增强综合生产能力。加大脱贫攻坚力度，实现10万贫困人口脱贫。抓好农村环境整治，建设30个康居示范村，实施80个市级村庄整治工程，新建一批户用沼气池、畜禽养殖场沼气治理工程，建成农村沼气站10个。建立长效运行管理机制，巩固乡镇文化站、村文化室、农家书屋全覆盖建设成果。完善农村流通网络，大力推进家电、农机下乡，农家店村级覆盖率提高10个百分点。

进一步放开搞活农村经济。深化农村综合改革，完善农业经营体制，大力发展农民专业合作、土地股份合作组织，新增合作社100家以上，增加土地流转面积20万亩，新增农业规模经营面积15万亩。完成集体林权制度改革。创新发展农村金融，加大金融支农力度，引导金融单位在乡村设立分支机构，推进农村贷款组织和村镇银行建设，建立农业投资担保公司，推广高效设施农业保险。培育农民创业主体，加强劳动技能培训，促进农村劳动力就地就近转移。培育发展农村经纪人2000人，培训农民20万人，新增农村劳动力转移3万人。

五、深化改革开放，强化以开拓创新为取向的机制建设

坚持改革创新，破解体制机制难题。做好市县政府机构改革工作，加快职能转变，全面开展乡镇机构改革，规范机构设置，提高行政效能。完善目标考核制度，制定完善科学发展评价和推进沿海开发考核体系，进一步增强导向性。扎实开展医药卫生体制五项重点改革，整合卫生资源，组建市级医疗集团，加快医疗卫生事业发展，提高公共卫生服务水平。完成文化体制改革任务，推动经营性文化事业单位转企和公益性文化事业单位内部改制，培育壮大文化产业市场主体。启动事业单位岗位设置改革，实施绩效工资。完善国有企业管理体制，促进国有资本资产重组和产业整合。积极开展金融创新，争取成为省金融综合改革实验区，鼓励金融保险和证券等机构创新金融产品，推进企业上市和上市再融资。着力整合社会资源引导创新创业实践，实施中小企业融资担保百亿工程，开展集成式社会化服务，增强民营经济和中小企业参与市场竞争、增加就业的活力和竞争力。建设小企业创业示范基地10家，新增私营企业6000家，个体工商户3万户。

加快开发开放，汇聚优质要素资源。强化载体功能建设，推动开发区建设从基础开发向完善生产服务配套转变，全面提升开发区建设发展水平。启动省级开发区申报扩区工作，积极申报国家级高新技术开发区，加快出口加工区、国际服务外包示范区等特色园区建设，实现保税物流中心一期封关运作，新增省级特色园区、南北共建园区2家。创新招商方式，加快政策推介型招商向产业营销型招商转变，举办新丝绸之路企业合作国际论坛，突出抓好67个投资3000万美元或5亿元以上的年度重大招商项目。强化综合协调服务，加大对30家重点外贸企业、100家出口增长企业的跟踪指导，继续抓好出口基地和品牌建设，支持企业开展境外技术合作和市场拓展，外贸出口增长10%，国际服务贸易增长20%。

六、推动和谐发展，致力以改善民生为重点的社会建设

更加注重就业和社会保障，着力富民惠民。加强职业技能培训和公共就业服务，实施特别职业培训计划，建立城乡一体的公共就业技能培训平台，加大对就业困难人员、零就业家庭、返乡农民工就业援助力度，城镇新增就业4.5万人。加快完善社会保障体系，推动社会保险向服务业、个体从业人员扩面覆盖，按照人均增长10%的标准，继续提高企业退休人员基本养老金水平，开通与上海异地就医结算。全面推进农村社保体系建设和被征地农民基本生活保障工作，畅通新老农保转换和城乡养老保险转移通道，进一步提高城乡基本社会保险覆盖率。促进房地产市场健康发展，增加普通住房供给，加强保障性住房建设，新建经济适用住房39万平方米、廉租住房773套，提供人才安置房100套。完善以城乡低保为主体的新型社会救助制度，实现对低收入群体全覆盖。加快示范性养老机构建设，落实尊老金补助和老人优待政策，改善五保老人生活待遇，提高社会福利保障水平。

更加注重社会事业协调发展，改善公共服务。坚持教育优先，促进均衡协调发展，学前教育毛入学率稳定在94%以上，小学、初中入学率均达100%，高中段教育毛入学率稳定在95%以上，省三星级以上学校就读比例达85%，高等教育毛入学率进一步提高。促进高等院校内涵建设，提升办学水平和层次。切实保障义务教育经费，确保经济困难家庭子女、进城务工人员子女、农村留守儿童平等接受义务教育。进一步改善办学条件，加快推进中小学校舍安全工程。大力发展城乡公共卫生，健全卫生监督体系，新型农村合作医疗参合率稳定在98%以上，住院费用实际报销比例进一步提高。农村卫生服务体系健全率、城市社区卫生服务人口覆盖率均达98%以上。开展第六次人口普查，在全省率先实现人口计划生育城乡优质服务体系全覆盖，人口自然增长率控制在7‰左右。繁荣发展文化事业，完善公共文化服务体系，筹办国际西游记文化旅游节。认真贯彻落实全民健身条例，增强群众体质，常年参加体育活动人群保持在50%以上，力争在十七届省运会上取得好成绩。为进一步提高公共产品供给能力，今年将重点推进一批社会事业项目。竣工东部城区连云港高级中学，建成苍梧小学，启动科教创业园区中小学建设。市一院综合楼投入使用，加快实施东方医院病房楼工程，启动建设市公共卫生中心。加快推进广播影视文化城、连云区文化创业中心建设。完成市体育中心主体育场建设、跳水游泳馆主体封顶、体育馆投入使用，四县体育中心基本建成。推进革命纪念馆新馆及陇海公寓复建工程。完成市老年大学搬迁改造，加快实施市社会福利中心工程，启动老干部活动中心建设。

更加注重精神文明和民主法制建设，完善社会管理。深化文明城市创建，广泛开展群众性精神文明活动，加强社会主义核心价值体系和公民道德建设，塑造文明连云港、诚信连云港、平安连云港形象。积极开展双拥工作，加强国防教育和动员，提高优抚安置保障水平，加强经济社会发展统计监测。做好民族宗教工作，抓好外事、侨务、对台事务，重视发展妇女儿童、残疾人、红十字、老区开发和关心下一代事业，推动气象、地方志、防震减灾等工作再上新台阶。完善质量安全监管机制，保障群众饮食用药安全。健全生产安全监控体系，实行企业安全标准化，促进安全生产形势持续稳定。推进乡镇依法行政和村务民主管理，做好村民委员会换届选举工作，开展和谐社区创建，建立社会稳定风险评估和信访工作代理机制，深入做好矛盾纠纷排查化解，社会矛盾纠纷调处成功率达95%以上。加强法治城市建设，构建完善的社会治安防控体系，万人刑事案件发案率控制在0.46%以下，提高突发公共事件应急处置能力，增强群众安全感。

七、提高行政效能，加强以人民满意为标准的政府建设

优化政府服务，增强发展推动力。进一步强化为企业发展服务，坚持有求必应、无事不扰，助企业创新创业，给企业雪中送炭。注重服务方式创新，加强规划引领、政策指导、信息服务，完善扶持产业发展、推进项目建设的措施办法。认真组织编制十二五规划。加强四级便民服务网建设，提升12345政府服务热线、市长信箱、群众来信来访办理服务质量。建立审批事项清理和流程优化动态机制，认真清理行政事业性收费，努力使连云港成为政府效能最高、行政成本最低的城市之一。

狠抓工作落实，提升行政执行力。强化调查研究，进一步构建科学民主的决策机制，主动接受人大法律监督、工作监督，积极支持人民政协履行政治协商、民主监督、参政议政职能，认真听取各民主党派、工商联、群团组织、专家学者和方方面面的意见，扎实办理人大代表议案、建议和政协委员提案，使政府的工作部署、政策措施，更加符合客观实际和群众意愿。创新重大项目领导办公会、难题会办会等机制，建立政府常务会议听取目标任务责任单位工作汇报制度，加大重点工作、重大事项和重要民生问题的跟踪落实力度。树立高效务实的工作导向，强化绩效考核，加强效能监察、行政问责。

加强作风建设，提高政府公信力。深化政务公开，加强政府新闻发布工作，扎实推进行政权力网上公开透明运行，建立完善电子监察系统，加强对政府性资金和投资项目的审计监督。大力发扬艰苦奋斗、勤俭办事作风，严格财政预算编制执行，严控出国出访考察和会议、接待、差旅等公务支出，实行一般性行政经费零增长，将有限的财力更多地投向创新创业和社会民生事业。进一步强化机关软环境建设，坚持勤政廉政，坚决反腐倡廉，努力打造高效务实、为民清廉的公务员队伍。

各位代表，连云港发展已经迎来加速崛起的重要机遇期。新的形势催人奋进，新的征程任重道远。让我们紧密团结在以胡锦涛同志为总书记的党中央周围，深入实践科学发展观，在中共连云港市委的领导下，以克难奋进的勇气、创新创造的举措、团结拼搏的作风，满怀激情，开拓进取，为加快昂起江苏沿海开发的龙头，推进全面跨越、后发先至而努力奋斗！
